# Predictive factors for 24 weeks sustained virologic response (SVR24) and viral relapse in patients treated with simeprevir plus peginterferon and ribavirin

**DOI:** 10.1007/s12072-015-9654-9

**Published:** 2015-08-12

**Authors:** Masahiko Nakayama, Hisanori Kobayashi, Koji Fukushima, Miwako Ishido, Yuji Komada, Kazutake Yoshizawa

**Affiliations:** Medical Affairs Division, Janssen Pharmaceutical K.K., 5-2, Nishi-kanda, 3-Chome, Chiyoda-ku, Tokyo, 101-0065 Japan; Research and Development Division, Janssen Pharmaceutical K.K., 5-2, Nishi-kanda, 3-Chome, Chiyoda-ku, Tokyo, 101-0065 Japan

**Keywords:** Hepatitis C, Simeprevir, Predictive factor, Viral relapse, Ribavirin dose intensity

## Abstract

**Background:**

Simeprevir with peginterferon and ribavirin has been used for the treatment of chronic hepatitis caused by genotype 1 hepatitis C virus (HCV). We explored the predictive factors for sustained virological response (SVR) and viral relapse using datasets from four Japanese phase 3 studies (CONCERTO).

**Methods:**

We used a multiple logistic regression model. First, an integrated dataset comprising 357 patients was analyzed. Subsequently, prior treatment-naïve and relapser (223 patients) and nonresponder (134 patients) of interferon-based treatment subsets were analyzed to identify predictors of SVR. A subset of nonresponders (106 patients) who were treated ≥24 weeks was also analyzed to identify predictors for viral relapse.

**Results:**

In the integrated dataset, prior treatment response was significantly associated with SVR. In subset analyses, interleukin-28B (IL28B) TT genotype and undetectable plasma HCV RNA level at week 4 were associated in treatment-naïve patients and relapsers [odds ratio (OR); 4.106 and 3.701, respectively]. In the nonresponders, the IL28B TT genotype population was very small, and inosine triphosphatase (ITPA) and undetectable plasma HCV RNA at week 4 were associated (OR; 2.506 and 3.333, respectively). Furthermore, ribavirin dose intensity (RBV-DI) and detectable plasma HCV RNA at week 4 were significantly associated with viral relapse (OR; 0.327 and 2.922, respectively).

**Conclusion:**

IL28B and plasma HCV RNA level at week 4 were clinically relevant predictive factors for SVR in treatment-naïve patients and relapsers. Moreover, RBV-DI and plasma HCV level at week 4 were identified as relevant predictive factors for viral relapse in nonresponders.

## Introduction

Hepatitis C virus (HCV) infection is one of the major concerns for public health, with approximately >180 million infections worldwide [1]. During the natural course of HCV infection, patients develop liver fibrosis, which gradually progresses to hepatocellular carcinoma in approximately 45 % of cases within 20 years from the initial infection [2]. Because interferons (IFNs) have been known to have potential eradication of the HCV, they have been used as the standard of care for HCV treatment [3, 4].

Sustained virological response (SVR), defined as undetectable HCV RNA by quantification assay at the end of treatment and 12–24 weeks after the end of treatment, has been used to assess therapeutic efficacy. In global HCV treatment guidelines, SVR is recommended as the goal of anti-HCV treatment, patients who achieved SVR experienced numerous benefits including a reduction of liver fibrosis and hepatocellular carcinoma [5, 6].

Over the past decade, the combination of peginterferon (PegIFN) plus ribavirin (RBV), developed for genotype 1 (GT1) HCV-infected patients, has conferred SVR rate of approximately 40–50 % for treatment-naïve GT1 HCV patients with high viral load [7, 8]. The limited SVR rates observed with this drug combination reiterated the importance of predictive factors for SVR when identifying appropriate patients that would benefit from this combination treatment, thereby necessitating the development of a new active agent [9–11]. Telaprevir is the first-generation oral NS3/4A protease inhibitor which was developed in combination with PegIFN plus RBV for its superior treatment efficacy. The SVR rates at 24 weeks after the end of treatment (SVR24) in treatment-naïve as well as prior IFN-based therapy relapser and nonresponder GT1 HCV patients were approximately 75, 83, and 41 %, respectively [12, 13].

Simeprevir [100 mg once daily (QD)] is a second-generation oral NS3/4A protease inhibitor, which was developed in combination with PegIFN and RBV. Four phase 3 studies (CONCERTO) were conducted in Japan. The SVR24 rates were 88.6 and 91.7 % for treatment-naïve; 35.8, 50.9, and 38.5 % for nonresponders; and 89.8 and 96.6 % for relapser patients [14–16]. Because simeprevir has favorable efficacy without inducing severe dermatologic and hematologic toxicity, it provides a better therapeutic index in GT1 HCV patients. Unfortunately, certain treatment failures have been reported: SVR24 was not favorable in nonresponder patients even after the achievement of an early viral response, and approximately 40 % of patients exhibited relapse after discontinuation of therapy [15]. The present study aimed to identify potential predictive factors for SVR24 and viral relapse to determine the patients most suitable to receive the combination of simeprevir with PegIFN and RBV by using a consolidated data set from the aforementioned CONCERTO studies.

## Methods

### Data source

The data for this post hoc study was derived from four phase 3 studies to assess the efficacy and safety of simeprevir plus PegIFN and RBV for the treatment of GT1 HCV-infected patients (CONCERTO-1, NCT01292239; CONCERTO-2, NCT01288209; CONCERTO-3, NCT01290731; and CONCERTO-4, NCT01366638). All patients who received simeprevir were included in this study. Original results from each CONCERTO study are available in the literature [14–16].

The CONCERTO studies included the following target population: CONCERTO-1, treatment-naïve patients (naïve); CONCERTO-2, prior nonresponders to IFN-based therapy (nonresponders); CONCERTO-3, prior relapsers to IFN-based therapy (relapsers); and CONCERTO-4, naïve, nonresponders, and relapsers. The eligibility criteria among the four studies were as follows: age between 20 and 70 years, chronic GT1 HCV infection, plasma HCV RNA levels ≥5.0 log_10_ IU/mL at screening, and no evidence of hepatic cirrhosis or hepatic failure. Dosage and treatment schedules in the CONCERTO-1,-2, and -3 studies were as follows: simeprevir 100 mg QD (53 CONCERTO-2 patients received this drug for 24 weeks) combined with PegIFN α-2a (180 μg once weekly) and RBV (total daily dose, 600–1000 mg/day, depending on body weight) for 12 weeks, followed by PegIFN α-2a and RBV alone for 12 weeks. PegIFN α-2b (1.5 μg/kg once weekly) was administered instead of PegIFN α-2a in CONCERTO-4. At week 24, patients either stopped or continued treatment with PegIFN and RBV according to response-guided therapy (RGT) criteria, except the CONCERTO-4 nonresponders. Patients who met the RGT criteria (i.e. achievement of HCV RNA levels <1.2 log_10_ IU/mL, either detectable or undetectable at week 4 and undetectable HCV RNA at week 12) could discontinue the therapy at week 24.

The primary endpoint of the original studies was SVR12, defined as the proportion of patients with undetectable HCV RNA at the end of treatment and at 12 weeks after the last dose of treatment, and the secondary endpoint was SVR24.

Because heterogeneity against SVR24 was strongly considered during prior treatment response, the dataset was divided into two subsets, naïve-relapsers and nonresponders, each of which was then individually analyzed. The analysis was first conducted using all integrated patient datasets, followed by the separate analysis of the two subsets divided based on prior treatment response.

### Available parameters

SVR24 and viral relapse were used as outcome variables in this study. Although SVR12 was the primary endpoint for the CONCERTO studies, we used SVR24 as the outcome variable in this analysis because it is more commonly used as a primary efficacy parameter.

Body weight, height, hematology, blood chemistry, genotype of interleukin-28B (IL28B) and HCV GT1 amino acid substitutions in the NS3 region were assessed at baseline. Baseline blood samples were collected from patients who consented to undergo exploratory host DNA genotyping. The HCV NS3 region was genotyped by using standard population sequence. Inosine triphosphatase (ITPA) was genotyped by using the Invader Plus assay. Plasma HCV RNA quantification assay was performed from screening to week 72 (patients stopping PegIFN and RBV at week 24) by using the Roche COBAS^®^ TaqMan^®^ HCV Auto assay system (lower limit of quantification, 1.2 log_10_ IU/mL).

To select independent variables, we examined the following characteristics which were considered clinically relevant to SVR24 and viral relapse: previous treatment response (naïve patients, relapsers, nonresponders); age; sex; baseline body mass index (BMI, kg/m^2^); baseline HCV RNA level (log_10_ IU/mL); baseline platelet count (×10^9^/L); baseline low density lipoprotein (LDL) cholesterol level (mmol/L); alpha-fetoprotein level (pmol/L); baseline fibrosis-4 (FIB-4) index (calculated as (age[year] × AST [U/L])/([PLT {10^(9)^/L}] × [ALT {U/L}]^(1/2)^); baseline hemoglobin (g/dL); IL28B genotype (rs8099917; TT, TG, and GG); ITPA genotype (rs1127354; CC, CA, and AA); and plasma HCV RNA level (detectable or undetectable) at weeks 2 and 4.

Type of PegIFN, cumulative dose of simeprevir (mg/kg), minimum hemoglobin level (mg/dL), and RBV dose intensity [RBV-DI; calculated as cumulative dose of RBV (mg)/treatment period (week)/body surface area (m²)] were included to the analysis for nonresponders.

Furthermore, cumulative dose of RBV (g/kg) was included in the analysis of viral relapse in the nonresponders who treated ≥24 weeks.

### Statistical analysis

Univariate and multivariate analyses were conducted to identify the potential predictors of SVR24 and viral relapse. Univariate analyses were sequentially performed for each independent variable to identify potential factors for further multivariate analysis. Fisher’s exact test and logistic regression models were used for comparisons between individual categorical independent variables and continuous variables, respectively. Variables with *p* values of <0.10 in the univariate analyses were included in the multivariate logistic regression analysis, which was performed using stepwise (backward) regression, and analyzed again for statistical significance. A *p* value of <0.05 was considered significant.

Receiver operating characteristic (ROC) curves were constructed for each logistic model, and the areas under the ROC (AUROC) curves were evaluated for predictive performance analysis. The 95 % confidence interval (CI) of the AUROC was computed by resampling 10,000 times (bootstrapping). The sensitivity and specificity of the ROC curves were determined at the cutoff point where (sensitivity + specificity − 1) it was maximal in the ROC curve (Youden index). ROC analysis was performed using the full model (including all statistically significant independent variables) as well as the limited model (each independent variable).

All statistical analyses were performed using R version 3.1.0 (a language and environment for statistical computing. R Foundation for Statistical Computing, Vienna, Austria. URL http://www.R-project.org/).

## Results

### Baseline demographics and clinical characteristics

In total, the CONCERTO studies enrolled 424 patients, of whom, 357 were eligible for this study. Specifically, 123, 106, 49, and 79 patients were from CONCERTO-1, -2, -3, and -4, respectively.

Baseline polymorphism on the HCV NS3 region was available from 353 patients, only five patients (1.4 %) presented Q80K and five patients (1.4 %) presented D168E polymorphism. The ITPA genotyping results were available for 308 patients. Patient demographics and baseline characteristics are summarized in Table 1.

**Table 1:** Patient demographics and baseline characteristics

Characteristic	Total	Naïve/relapser	Nonresponder	Nonresponder treated ≥24 weeks
	*n* = 357	*n* = 225	*n* = 132	*n* = 106
Age, years, median (range)	59 (22–70)	59 (22–70)	60 (24–70)	59 (24–70)
Sex				
Male/female	149/208	83/142	66/66	48/58
BMI, kg/m², median (range)	22.4 (16.8–34.3)	22.4 (16.9–32.9)	22.25 (16.8–34.3)	22.15 (16.8–33.4)
HCV RNA, log_10_ IU/mL, median (range)	6.4 (4.5–7.4)	6.4 (4.5–7.4)	6.5 (4.6–7.4)	6.5 (4.6–7.3)
platelet count (×10^9^/L), *n* median (range)	168 (90–392)	175 (95–333)	161 (90–392)	164 (92–330)
LDL cholesterol (mol/L) median (range)	2.79 (0.90–6.18)	2.90 (1.37–6.18)	2.50 (0.91–4.769)	2.56 (0.91–4.769)
Alpha-fetoprotein (ng/mL) median (range)	63.51 (17.57–885.10)	59.46 (17.57–504.00)	72.29 (20.27–885.10)	72.97 (20.27–885.10)
FIB-4 index median (range)	1.98 (0.28–8.29)	1.82 (0.28–8.29)	2.31 (0.42–7.76)	2.21 (0.43–6.78)
Hemoglobin (mg/dL) median (range)	14.0 (11.1–17.9)	13.9 (11.1–17.9)	14.2 (11.4–16.9)	14.2 (11.4–16.9)
IL28B genotype (rs8099917), *n* TT/TG + GG	175/182	159/66	16/116	8/98
ITPA genotype (rs1127354), *n* CA + AA/CC/not available	89/219/49	51/144/30	38/75/19	35/56/15
HCV genotype 1a/1b	7/350	3/222	4/128	3/103
HCV with baseline Q80K (%)	5/353 (1.4 %)	4/221 (1.8 %)	1/132 (0.8 %)	0/106
HCV with baseline D168E (%)	5/353 (1.4 %)	4/221 (1.8 %)	1/132 (0.8 %)	0/106
Patient number from CONCERTO-4	79	53	26	21

*ITPA* inosine triphosphatase, *BMI* body mass index, *IL28B* interleukin-28B, *LDL* low density lipoprotein

### Study medication

Table 2 presents information regarding completion and discontinuation of study treatment and results of efficacy endpoints for each subset. Treatment-naïve patients and relapsers were successfully treated with simeprevir and PegIFN plus RBV, with a treatment completion rate of 93.8 %. Except one patient, all patients completed treatment, met RGT criteria and finished study treatment at 24 weeks. On the other hand, the nonresponders exhibited less unfavorable completion rates, with 75.0 % of patients completing the planned treatment. Eighty-two (77.4 %) of 106 patients in CONCERTO-2 met RGT criteria, and 16 of 26 patients were treated ≥24 weeks of treatment in CONCERTO-4. The main reason for discontinuation (>5 %) was the virologic stopping criteria; 13.6 % of the patients discontinued the treatment because of these criteria.

**Table 2:** Treatment exposure status of study medication and efficacy endpoints in each study

Treatment exposure	Naïve/relapser	Nonresponder	Nonresponder treated ≥24 weeks
Complete all study medicine^a^ (%)	211/225 (93.8)	99/132 (75.0)	99/106(93.4)
Study medication discontinuation (%)	14/225 (7.3)	33/132 (25.0)	7/106 (6.6)
Withdrawal of consent	1 (0.8)	3 (2.2)	
Adverse event	10 (4.9)	5 (3.8)	1 (0.9)
Met the virologic stopping criteria^b^		18 (13.6)	6 (5.7)
Investigator’s judgment	1 (0.8)	3 (1.9)	
Other	2 (0.8)	4 (3.0)	
Met RGT^c^criteria (%)	210/225 (91.9)	82/106 (77.4)	82/85 (96.5)
Median cumulative dose of SMV (mg/kg)	147.8 (37.2–205.3)	163.8 (23.96–375.0)	175.9 (94.24–375.0)
Median cumulative dose of RBV(g/kg)	1.77 (0.22–4.15)	1.79 (0.22–4.41)	1.91 (0.99–4.41)
Median dose intensity of RBV (mg/week/m²)	–	2791 (1452–3698)	2763 (1452–3659)
Median SMV AUC_24 h_^d^ (ng h/mL)	C1: 42721, C4N: 35448 C3: 63261, C4R: 68130	C2: 62313, C4: 40645	–
Median SMV CL/F (L/h)	C1: 2.34, C4N: 2.83 C3:1.58, C4R:2.46	C2: 1.60, C4: 1.47	–
HCV RNA at week 2			
Detectable/undetectable	153/72	124/8	99/7
HCV RNA at week 4			
Detectable/undetectable	37/187	58/73	39/67
SVR12 (%)	207/225 (92.0)	57/132 (43.2)	56/106 (52.8)
SVR24 (%)	203/225 (90.2)	56/132 (42.4)	55/106 (51.9)

*SMV* simeprevir, *RBV* ribavirin, *CL/F* apparent clearance, *SVR12* sustained virologic response at week 12, *SVR24* sustained virologic response at week 24, *C1* CONCERTO-1,* C2* CONCERTO-2, *C3* CONCERTO-3, *C4* CONCERTO-4, *C4N* CONCERTO-4 naïve, *C4R* CONCERTO-4 relapser

^a^Completed all study medicine definitions: patients who finished study treatment according to RGT criteria or patients who completed study treatment at 48 weeks. RGT criteria was not adopted for nonresponders in CONCERTO-4

^b^Virologic stopping criteria: patients with suboptimal response discontinued treatment in a timely manner

^c^ *RGT* response-guided therapy criteria. If patients achieved HCV RNA detection <1.2 log_10_ IU/mL or no detection at week 4, with no detection of HCV RNA at week 12, peginterferon plus ribavirin therapy could be stopped at week 24. RGT criteria was not adopted for nonresponders in CONCERTO-4

^d^Area under the plasma concentration–time curve (from 0 to 24 h)

### Predictive factor analysis results

#### Integrated analysis of all patient datasets

**Fig. 1:**
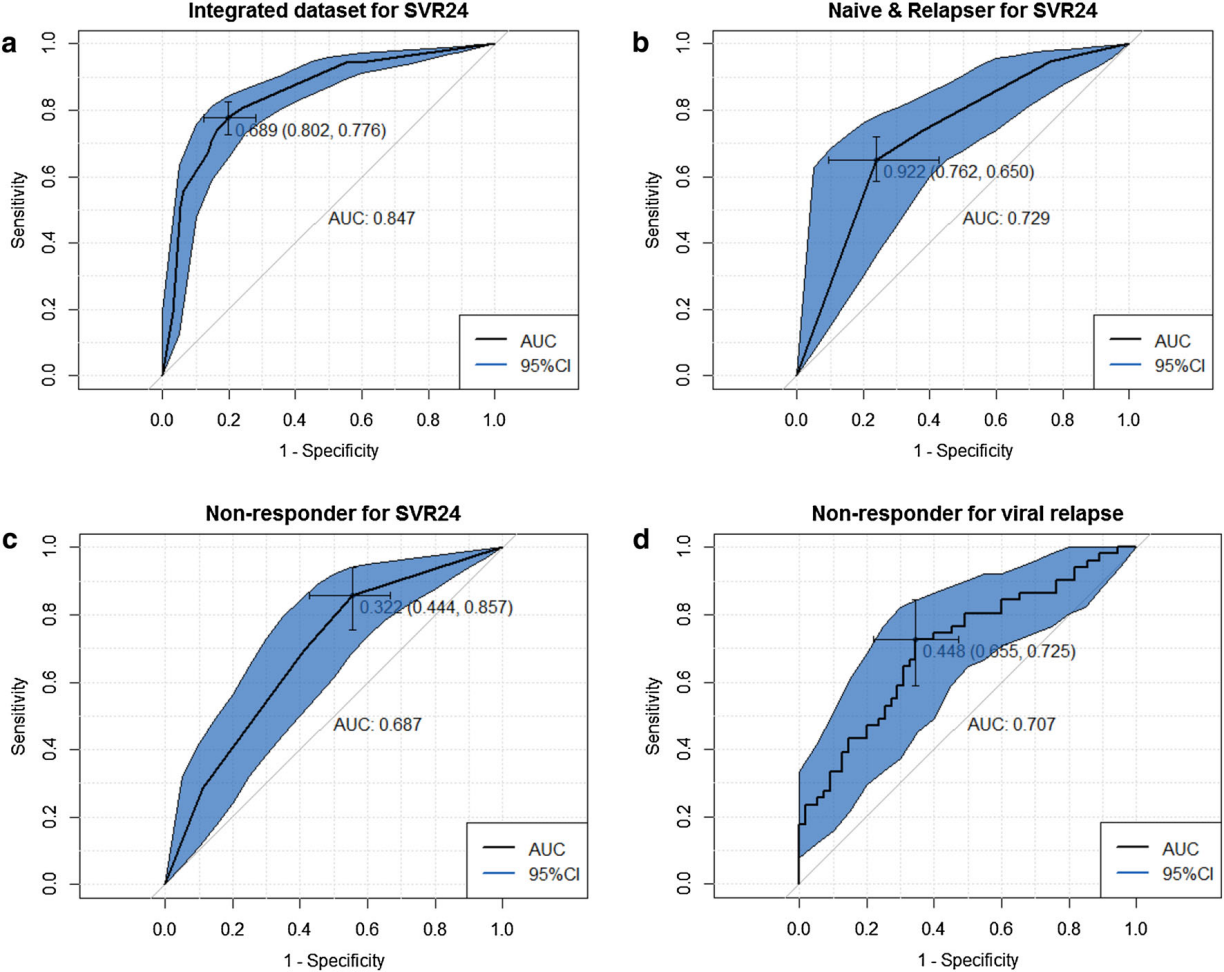
ROC curves of full models in the (**a**) integrated dataset for SVR24 (*n* = 357), (**b**) subset of naïve patients and relapsers for SVR24 (*n* = 225), (**c**) subset of nonresponders for SVR24 (*n* = 132), and (**d**) subset of nonresponders who completed 24 weeks of treatment for viral relapse (*n* = 106).* AUC* areas under the curve,* 95 % CI* 95 % confidence interval

Eight variables revealed significant association with SVR24 in the integrated dataset of 357 patients using the univariate analyses. Subsequent multivariate analysis identified prior treatment responders and IL28B genotypes as significant predictors of baseline characteristics. The odds ratios (ORs) of prior treatment responses in naïve patients versus nonresponders and in relapsers versus nonresponders were 6.298 (95 % CI 3.149–13.192, *p* < 0.001) and 7.731 (95 % CI 3.011–22.845, *p* < 0.001), respectively (Table 3). The AUROC was 0.847 (95 % CI 0.798–0.889) in the full model (Fig. 1a).

**Table 3:** Factors associated with SVR24: univariate and multivariate analyses (using patient data from all four CONCERTO studies)

Factor	Univariate analysis			Multivariate analysis		
	OR	95 % CI	*p* value	OR	95 % CI	*p* value
Sex						
Female	Ref					
Male	1.006	0.609–1.652	>0.999			
Age	0.986	0.961–1.010	0.265			
Prior treatment response						
Nonresponder	Ref					
Naïve	11.111	6.094–21.322	<0.001	6.298	3.149–13.192	<0.001
Relapser	16.286	7.101–44.347	<0.001	7.731	3.011–22.845	<0.001
BL LDL-cholesterol (mmol/L)	1.949	1.388–2.794	<0.001			
BL alpha-fetoprotein (pmol/L)	0.997	0.994–0.999	0.014			
IL28B genotype (rs8099917)						
TG/GG	Ref					
TT	6.803	3.777–12.803	<0.001	2.490	1.206–5.213	0.014
ITPA genotype (rs1127354)						
CC	Ref					
CA/AA	1.423	0.778–2.683	0.260			
BL platelet count (×10^9^/L)	1.006	1.001–1.012	0.022			
BL hemoglobin (g/dL)	1.005	0.987–1.024	0.587			
BL BMI (kg/m²)	1.026	0.947–1.115	0.537			
BL FIB-4 index	0.744	0.611–0.902	0.003			
BL HCV RNA (log_10_ IU/mL)	0.701	0.433–1.113	0.1391			
HCV RNA at week 2						
Detectable	Ref					
Undetectable	7.550	2.949–24.748	<0.001			
HCV RNA at week 4						
Detectable	Ref					
Undetectable	5.505	3.198–9.575	<0.001	3.589	1.987–6.546	<0.001

*BL* baseline,* LDL* low density lipoprotein, *IL28B* interleukin-28B, *ITPA* inosine triphosphatase,* BMI* body mass index, *Ref* reference

#### Subset analysis according to prior treatment response: naïve patients and relapsers

A total of 225 naïve patients and relapsers from CONCERTO-1, -3, and -4 were subjected to subset analysis. In the IL28B genotype, 70.7 % (159/225) of patients were TT genotype in this subset. The multivariate analysis showed that still the IL28B genotype was a baseline predictive factor. The OR of IL28B TT versus TG + GG was 4.106 (95 % CI 1.601–11.046, *p* = 0.004). Undetectable plasma HCV RNA at week 4 was identified as a treatment-associated variable, with an OR of 3.071 (95 % CI 1.089–8.241, *p* = 0.028; Table 4). Figure 1b shows the ROC curve of the full model, with an AUROC of 0.729 (95 % CI 0.6146–0.8327).

**Table 4:** Factors associated with SVR24 in naïve patients and relapsers: univariate and multivariate analyses

Factor	Univariate analysis			Multivariate analysis		
	OR	95 % CI	*p* value	OR	95 % CI	*p* value
Sex						
Female	Ref					
Male	2.115	0.712–7.631	0.169			
Age	0.997	0.951–1.040	0.906			
Prior treatment response						
Naïve	Ref					
Relapser	1.463	0.515–4.773	0.491			
BL LDL-cholesterol (mmol/L)	1.560	0.853–3.018	0.167			
BL alpha-fetoprotein (pmol/L)	0.996	0.990–1.002	0.134			
IL28B genotype (rs8099917)						
TG/GG	Ref					
TT	4.058	1.506–11.431	0.002	4.106	1.601–11.046	0.004
ITPA genotype (rs1127354)						
CC	Ref					
CA/AA	1.466	0.442–6.334	0.601			
BL platelet count (×10^9^/L)	1.009	0.998–1.021	0.139			
BL hemoglobin (g/dL)	0.970	0.967–1.038	0.969			
BL BMI (kg/m²)	1.126	0.955–1.355	0.184			
BL FIB-4 index	0.809	0.586–1.164	0.217			
BL HCV RNA (log_10_ IU/mL)	1.100	0.454–2.507	0.825			
HCV RNA at week 2						
Detectable	Ref					
Undetectable	3.247	0.909–17.719	0.057			
HCV RNA at week 4						
Detectable	Ref					
Undetectable	3.663	1.206–10.552	0.011	3.071	1.089–8.241	0.028

*BL* baseline,* LDL* low density lipoprotein, *IL28B* interleukin-28B, *ITPA* inosine triphosphatase,* BMI* body mass index, *Ref* reference

#### Subset analysis according to prior response: nonresponders

In total, 132 nonresponders from CONCERTO-2 and -4 were analyzed. IL28B genotype was more distinct in this subset than that in the naïve and relapser subset, i.e. 116 (87.9 %) of 132 patients exhibited TG and GG genotypes (Table 1). On univariate analysis, the IL28B genotype failed to show significance for prediction. Multivariate analysis revealed the ITPA genotype as a baseline predictive factor, with an OR of 2.506 (95 % CI 1.096–5.907, *p* = 0.003). SVR24 rate for ITPA CC was 36.0 % (27/75) and 57.9 % (22/38) for non-CC. Among 26 patients who discontinued the therapy within 24 weeks, 22 patients were available for ITPA genotype. Nineteen patients had ITPA CC and 18 of them relapsed. Prediction accuracy for ITPA was not favorable; AUROC was 0.598 (95 % CI 0.508–0.684). Undetectable plasma HCV RNA at week 4, as a treatment-associated variable, was identified as a significant predictive factor, with an OR of 3.333 (95 % CI 1.509–7.666, *p* = 0.004) (Table 5). Figure 1c shows the ROC curve of the full model, with an AUROC of 0.687 (95 % CI 0.5896–0.7784).

**Table 5:** Factors associated with SVR24: univariate and multivariate analyses in nonresponders

Factor	Univariate analysis			Multivariate analysis		
	OR	95 % CI	*p* value	OR	95 % CI	*p* value
Sex						
Female	Ref					
Male	1.280	0.606–2.714	0.598			
Age	0.984	0.948–1.021	0.396			
Type of interferon						
IFN alpha-2b	Ref					
IFN alpha-2a	1.225	0.471–3.321	0.825			
BL LDL-cholesterol (mmol/L)	1.179	0.698–2.005	0.538			
BL alpha-fetoprotein (pmol/L)	1.000	0.997–1.002	0.772			
IL28B genotype (rs8099917)						
TT	Ref					
TG/GG	1.062	0.313–3.461	>0.999			
ITPA genotype (rs1127354)						
CC	Ref					
CA/AA	2.424	1.022–5.877	0.029	2.506	1.096–5.907	0.032
BL platelet count (×10^9^/L)	1.003	0.997–1.010	0.347			
BL hemoglobin (g/dL)	1.029	1.001–1.059	0.045			
BL BMI (kg/m²)	0.997	0.887–1.118	0.960			
BL FIB-4 index	0.784	0.564–1.058	0.126			
BL HCV RNA (log_10_ IU/mL)	0.493	0.240–0.980	0.048			
HCV RNA at week 2						
Detectable	Ref					
Undetectable	4.391	0.747–46.186	0.071			
HCV RNA at week 4						
Detectable	Ref					
Undetectable	3.635	1.640–8.375	0.001	3.333	1.509–7.666	0.004
Minimum hemoglobin (g/dL)	1.092	0.848–1.412	0.493			
Cumulative dose simeprevir (mg/kg)	1.003	0.998–1.007	0.249			
Ribavirin dose intensity (mg/week/m²)	1.616	0.771–3.483	0.209			

*BL* baseline,* LDL* low density lipoprotein, *IL28B* interleukin-28B, *ITPA* inosine triphosphatase,* BMI* body mass index, *Ref* reference

#### Predictive factors for viral relapse in prior nonresponders

In CONCERTO-2, although 82 of 106 patients had met the RGT criteria, 36 of them (43.9 %) relapsed after treatment completion. So, we analyzed predictive factors for viral relapse in nonresponders. A subset of 106 nonresponders from CONCERTO-2 and -4, who were treated with ≥24 weeks of therapy, were selected for this analysis. In this subset, a total of 51 patients (48.1 %) relapsed beyond 24 weeks of the therapy. On univariate analysis ITPA genotype failed to show significance for prediction. Relapse rates were 53.6 % (30/56) and 37.1 % (13/35) for ITPA CC and non-CC, respectively.

**Fig. 2:**
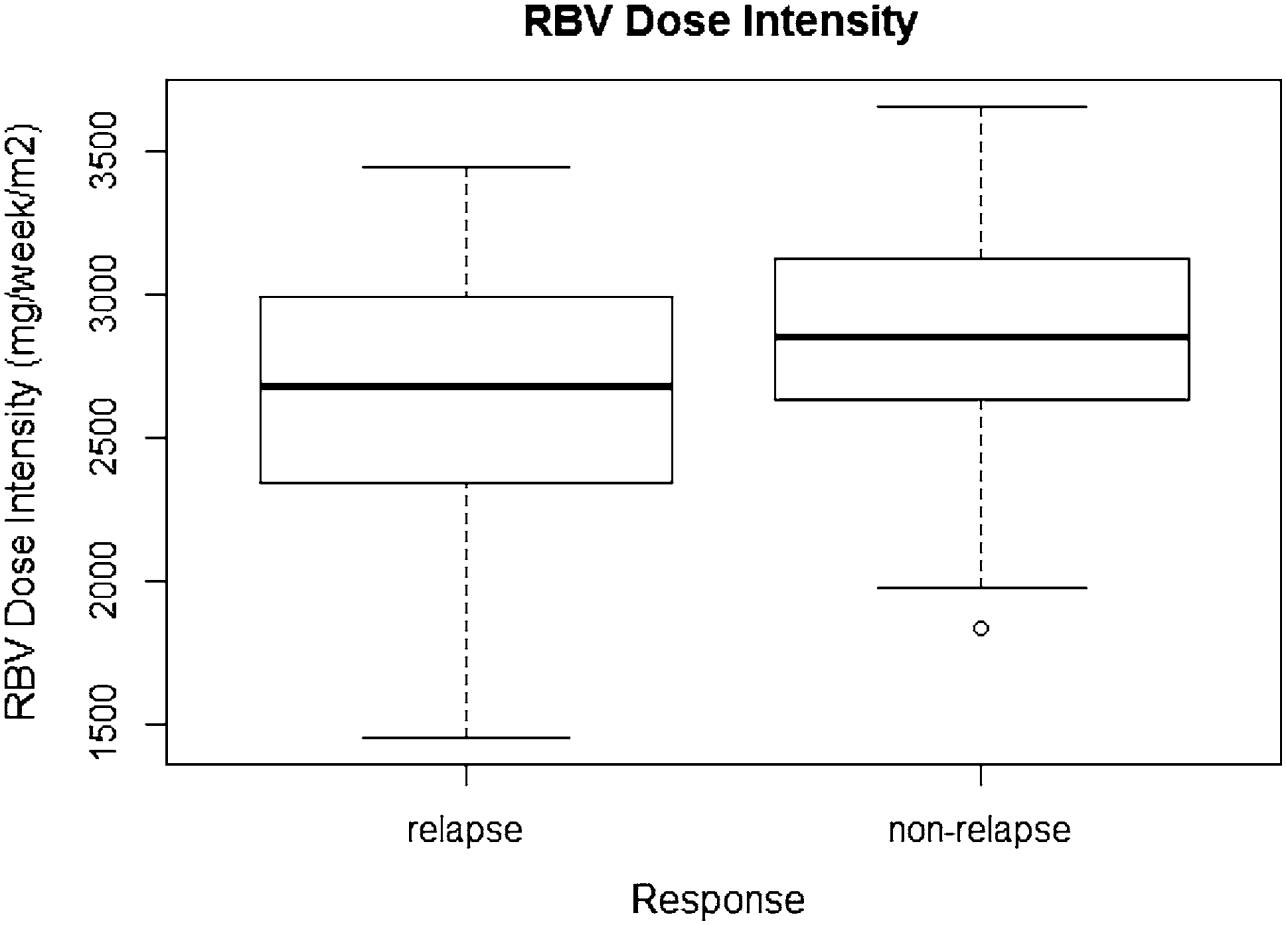
Box plot of the ribavirin dose intensity in subset of nonresponders who treated ≥24 weeks (*n* = 106)

Multivariate analysis identified baseline HCV RNA level as a significant baseline factor, with an OR of 2.536 (95 % CI 1.100–6.322, *p* = 0.036); however, median RNA levels were mostly overlapped, 6.4 log_10_IU/mL (range 4.6–7.1) for patients with SVR and 6.5 log_10_IU/mL (range 5.5–7.3) for patients without SVR. Its prediction accuracy was not favorable, AUROC was 0.591 (95 % CI 0.4825–0.697). Plasma HCV RNA detection at week 4 and RBV-DI were identified as treatment-associated variables, with ORs of 2.922 (95 % CI 1.255–7.079, *p* = 0.015) and 0.327 (95 % CI 0.121–0.823, *p* = 0.021), respectively (Table 6). The AUROCs for RBV-DI and plasma HCV RNA level at week 4 were similar: 0.612 (95 % CI 0.502–0.718) and 0.618 (95 % CI 0.530–0.705), respectively. Figure 2 shows the box plot of RBV-DI. The RBV-DI threshold value was 2,790 mg/week/m² as per the ROC cutoff point calculations. The viral relapse rates were 57.1 % (95 % CI 44.2–70.1) and 38.0 % (95 % CI 24.5–51.5) in patients with RBV-DI below and above the threshold, respectively.

**Table 6:** Factors associated with viral relapse: univariate and multivariate analyses in nonresponders who completed 24 weeks of treatment

Factor	Univariate analysis			Multivariate analysis		
	OR	95 % CI	*p* value	OR	95 % CI	*p* value
Sex						
Male	Ref					
Female	2.183	0.939–5.188	0.053			
Age	1.014	0.972–1.060	0.520			
Type of interferon						
IFN alpha-2a	Ref					
IFN alpha-2b	1.235	0.425–3.627	0.808			
BL LDL-cholesterol (mmol/L)	0.941	0.535–1.647	0.831			
BL alpha-fetoprotein (pmol/L)	1.001	0.997–1.003	0.751			
IL28B genotype (rs8099917)						
TT	Ref					
TG/GG	7.182	0.870–334.513	0.062			
ITPA genotype (rs1127354)						
CA/AA	Ref					
CC	1.938	0.759–5.099	0.138			
BL platelet count (x10^9^/L)	0.996	0.987–1.003	0.274			
BL hemoglobin (g/dL)	0.729	0.530–0.985	0.044			
BL BMI (kg/m²)	0.959	0.835–1.097	0.546			
BL FIB-4 index	1.172	0.824–1.693	0.381			
BL HCV RNA (log_10_ IU/mL)	2.335	1.064–5.458	0.041	2.536	1.100–6.322	0.035
HCV RNA at week 2						
Undetectable	Ref					
Detectable	6.037	0.693–286.728	0.114			
HCV RNA at week 4						
Undetectable	Ref					
Detectable	2.787	1.155–6.960	0.016	2.922	1.255–7.079	0.015
Minimum hemoglobin (g/dL)	0.793	0.562–1.099	0.171			
Cumulative dose simeprevir (mg/kg)	1.003	0.998–1.009	0.197			
Cumulative dose ribavirin (g/kg)	0.739	0.434–1.209	0.239			
Ribavirin dose intensity (mg/week/m²)	0.372	0.146–0.887	0.030	0.327	0.121–0.823	0.021

*BL* baseline, *ITPA* inosine triphosphatase, *Ref* reference

Figure 1d shows the ROC curve of the full model, with an AUROC of 0.707 (95 % CI 0.603–0.803).

## Discussion

Similar to previous reports [11, 17], SVR24 rates were higher in naïve patients and relapsers than in nonresponders in the CONCERTO studies. Based on these findings, we primarily analyzed the integrated patient data of four CONCERTO studies and confirmed the heterogeneity of nonresponders with regard to other patient subsets.

Analysis of the naïve patients and relapsers revealed the IL28B genotype as a predictive factor for SVR24. IL28B has been a well-known predictor for PegIFN plus RBV in GT1 HCV patients [18, 19]. Its predictability has been reported in combination with telaprevir or boceprevir [20, 21]. According to these findings, even after significant improvements in SVR24 rates following the use of NS3/4A protease inhibitors, IL28B was clinically confirmed as an important predictive factor for SVR24 with IFN-containing regimens. Especially for naïve and relapsers with IL28B TT, shorter treatment duration by RGT criteria is desirable to reduce the potential treatment related AEs and treatment cost.

No factors related to hepatic status were identified in the present study. Platelet counts and alpha-fetoprotein levels have been previously identified [21, 22]. In the CONCERTO studies, patients were selected by the inclusion criteria. However, in real-world settings, a variety of patients are treated with simeprevir and PegIFN plus RBV. Therefore, further analysis using data from clinical settings is necessary to confirm the results of this study.

Considering the difficulties in achieving SVR in the nonresponders, determination of the predictive factors for SVR and viral relapse is critical. Basically, in contrast to naïve and relapsers, unfavorable IL28B genotype was dominant in this subset.

Mutation in the HCV RNA might be a potential reason for unsatisfactory response. Unfavorable SVR rate was reported in simeprevir-treated HCV genotype 1a patients with baseline Q80 K [23, 24]. Because patients in CONCERTO studies were mostly genotype 1b, prevalence of Q80K was only 1 % by population sequencing. Recently, Akuta et al. [25] studied a simeprevir-resistant variant with ultra-deep sequencing, such an approach could reveal new findings. Besides, the core region was not evaluated in the CONCERTO studies, which has been shown as a predictive factor for SVR with telaprevir and PegIFN plus RBV in nonresponders [22].

Several factors had to be considered with regard to treatment exposure. First, although pharmacokinetics of simeprevir was evaluated using population pharmacokinetics in the CONCERTO studies, no significant differences were observed across all studies (Table 2). Because 83.7–88.0 % of patients achieved complete early virologic response (cEVR) in CONCERTO-2, low SVR rates in the nonresponders might not have been caused by simeprevir adherence and its associated pharmacokinetics. Second, several studies have reported the development of an anti-IFN antibody in 5–15 % of patients, with higher rates of development in patients previously treated with IFN [26–28]. Anti-IFN antibody was not evaluated in the CONCERTO studies; it might cause treatment failure. Last, even after the advent of protease inhibitors, SVR24 rate was remarkably lower without RBV, and it has been considered as an essential component of protease inhibitor containing triple therapy [29]. The relationship between viral relapse and RBV dosage has been studied previously, adequate RBV-DI (≥6 mg/kg/day) was recommended in patients treated with PegIFN plus RBV for 48 weeks to prevent virus relapse [30, 31]. In the present study, relapse rate was statistically lower (38.0 %) in patients with RBV-DI > 2790 mg/week/m² (approximately 11 mg/kg/day). Both relapse rate and threshold of RBV-DI were higher in our study compared with those reported in previous studies, although patient background, particularly prior treatment response, and treatment duration were different among these studies. Bodeau et al. [32] studied the relationship between plasma concentrations of RBV at the end of treatment (week 48) and viral relapse, they reported that low RBV plasma concentrations resulted in significantly higher relapse rates. Overall, viral relapse could be mediated by RBV plasma concentrations even after the addition of a protease inhibitor to PegIFN plus RBV. Moreover, Oze et al. [33] have reported that the relapse rate was significantly decreased in patients with longer treatment durations (72 weeks) than in those with shorter treatment durations (48 weeks). Because most nonresponders completed the treatment at 24 weeks by the RGT criteria, a major limitation of this post hoc study was the lack of adequate evaluation of optimal treatment duration. Further investigations are warranted to explore and determine the optimal treatment duration with PegIFN plus RBV in nonresponders to prevent viral relapse after achievement of early viral response.

In addition, ITPA was revealed as a predictor of nonresponders, however, not in the ≥24 weeks treated nonresponders. Since ITPA CC was a well-known risk factor for RBV induced anemia, discontinuation of the therapy or insufficient RBV dose could be induced more frequently on this population. Patients with early discontinuation were mainly ITPA CC patients and they were removed from the viral relapse analysis; we considered this would make a discrepancy in the results of ITPA.

A few limitations to our study should be noted. Since the present study was a post hoc study which utilized merged data from registration studies and original studies were not designed to analyze predictive factors, it involved the selection of patients who were physically healthy, which would limit the extrapolation of our results in real-world clinical settings. Thus, additional studies using different participants, particularly in clinical settings, are warranted to confirm these results and their generalizability as well as to identify other meaningful predictive factors.

In conclusion, our results have identified the following clinically relevant predictive factors for SVR; undetectable plasma HCV RNA at week 4 for all patients and IL28B for naïve and relapsers. For viral relapse, RBV-DI and plasma HCV RNA detection were identified as clinically relevant predictive factors in nonresponders who were treated for ≥24 weeks without suboptimal response.
